# Dynamic link between central bank reserves, credit default swap spreads, and foreign exchange rates: Evidence from Turkey by time series econometrics

**DOI:** 10.1016/j.heliyon.2023.e16392

**Published:** 2023-05-20

**Authors:** Mustafa Tevfik Kartal, Talat Ulussever, Ugur Korkut Pata, Serpil Kılıç Depren

**Affiliations:** a Borsa Istanbul Strategic Planning, Financial Reporting, and Investor Relations Directorate, İstanbul, Turkey; b Gulf University for Science and Technology Department of Economics and Finance, Hawally, Kuwait; c Research Fellow, Gulf University for Science and Technology Center for Sustainable Energy and Economic Development (SEED), Hawally, Kuwait; d Osmaniye Korkut Ata University Department of Economics, Osmaniye, Turkey; e Yildiz Technical University Department of Statistics, İstanbul, Turkey

**Keywords:** CBR, CDS Spreads, FX Rates, Time series econometrics, Turkey

## Abstract

In this study, dynamic links between central bank reserves (CBR), credit default swap (CDS) spreads, and foreign exchange (FX) rates are investigated. So, Turkey, which is a negative outlier country among other peer emerging countries, is examined by considering recent developments on these indicators. In doing so, the study covers relatively high frequency (i.e., weekly) data from January 2, 2004 to November 12, 2021, performs various econometric approaches as Wavelet Coherence (WC), Quantile-on-Quantile Regression (QQR), and Granger Causality in Quantiles (GCQ) as main models, and applies Toda-Yamamoto (TY) causality and Quantile Regression (QR) for the robustness. The results show that (i) there is a time-frequency dependency between the CBR, CDS spreads, and FX rates; (ii) a bidirectional link exists between the CBR and FX rates; between the FX rates and CDS spreads; and between the CDS spreads and CBR; (iii) the link exists in most quantiles except for some lower and middle quantiles for some indicators; (iv) explanatory effect of the indicators on each other varies based on quantiles; (v) the robustness of the results are validated by the TY causality test for the WC model and by the QR approach for the QQR model. The results suggest the significance of the CBR for the FX rates, the FX rates for the CDS spreads, and the CDS spreads for the CBR.

## 1 Introduction

All countries target to achieve economic growth and make economic growth sustainable [1]. On the other hand, countries can realize economic development through economic growth in the middle or long term. In this context, economic growth and development are such critical concepts to be followed and reflected by macroeconomic and financial indicators. These indicators are affected by the growth and development of economies as well. In other words, while macroeconomic and financial indicators are significantly important for reflecting economies’ growth, development, and well-being, the stability of these indicators is also important and plays a key role, which is effective both in real and financial sectors. Accordingly, the instability of those indicators can cause negative developments in economic stability, financial stability, and price stability [2]. Hence, it can be concluded that there is a bidirectional link between macroeconomic and financial indicators and financial markets [3,4]. Thus, the progress of such indicators has great importance for countries and economies.

In economies, there are a variety of macroeconomic and financial indicators. Economic growth, inflation, interest rates, credit/deposit ratio, credit growth, non-performing loans, stock market indices, and unemployment are typical examples of these macroeconomic and financial indicators [[5], [6], [7], [8], [9]]. Although there are many indicators, CBR, CDS spreads, and FX rates are the ones, which are the most important indicators reflecting the soundness, riskiness, vulnerability, and predictability of countries [[10], [11], [12]].

In globalizing economic relations and the financial connectedness of the world, the progress of the CBR, CDS spreads, and FX rates has been playing a much more important role. Any adverse development in these indicators can easily cause a deeply negative effect on economic, financial, and price stabilities. Negative progress can cause other internal and external economic and financial shocks as well [[13], [14], [15]]. For this reason, countries should monitor strictly and direct carefully these indicators to effectively prevent their adverse effects. The CBR has an important role in international payments and stabilizing FX rates because countries can use CBR frequently in balancing the value of national currencies against FX rates [13]. Also, CDS spreads are significant for countries since they are effective on foreign portfolio flows due to the insurance function and can be used country risk indicator [16]. Moreover, FX rates are quite crucial because a big share of international economic activities are realized with FX payments rather than national currencies [17].

Most of the developed countries have a generally stable trend in these indicators, whereas some emerging countries have a highly fluctuating trend [18]. Annex 1 shows the recent progress of CDS spreads by representing how many countries risk change in emerging countries that include most of the BRICST, Emerging-7, MINT, and Fragile Five countries. As Annex 1 presents, the CDS spreads of emerging countries have been generally unstable over time. Specifically, Turkey and South Africa are two countries that have generally higher CDS spreads than other peer-emerging ones. It can further be stated that Turkey has the highest and most volatile CDS spreads among all other peer countries. Moreover, Annex 2 presents the recent progress of the value of national currencies against the United States Dollar (USD) that shows by representing how much FX rates change in emerging countries against USD over time from 2016 January to 2021 October. As is seen in Annex 2, the value of the national currencies of emerging countries except for Turkey has been somehow stable over time. The value of the Turkish Lira (TRY) has been decreasing by a significant amount over recent years implying how much FX rates (proxied by USD) have been increasing. Furthermore, the outlook of the CBR is very similar among these emerging countries. The progress of the CBR is not good for Turkey as it is reflected in economic news and reports [19,20]. In conclusion, Turkey is considered a negative outlier country among peer countries in terms of the progress of these indicators.

In the literature, various studies scrutinize CBR [15,[21], [22], [23], [24], [25]], CDS spreads [10,11,16,[26], [27], [28]], and FX rates [12,26,[29], [30], [31]]. Even, some studies examine some of these indicators with other indicators in the same study. For example [30], examines FX rates and sovereign risk, whereas [24] investigates CBR and sovereign risk. However, there is no study in the literature, which inspects the dynamic link between CBR, CDS spreads, and FX rates simultaneously. Likewise, link between the indicators has not been explored for Turkey.

When evaluating the above-explained information altogether, it can be stated that (i) there is no study in the literature, which examines the dynamic link between CBR, CDS spreads, and FX rates; (ii) Turkey has adverse progress for the CBR, CDS spreads, and FX rates. Thus, an empirical analysis that focuses on Turkey can provide insights for the examination of a dynamic link between indicators especially by using high-frequency data and performing various time series econometrics models. Naturally, a variety of econometric techniques, such as vector autoregressive and panel data models, are used in the literature. Moreover, different data frequencies either low-frequency data as monthly and weekly [16,24,30] or high-frequency data as daily [27] are used for the examination of the aforementioned indicators. Hence, such a specific study fixes the literature gap and makes contributions.

The study aims to investigate the dynamic link between the aforementioned indicators in an outlier emerging country example, which has high volatility and adverse progress in these indicators. More specifically, by including the indicators of CBR, CDS spreads, and FX rates, covering the weekly data from January 2, 2004 to November 12, 2021, which is a long enough period, and performing various econometric approaches (WC, QQR, and GCQ), the study analyzes power impact and causal impact for Turkey. Moreover, TY and QR approaches are applied for the robustness. All in all, the study tries to answer the following questions; (i) do changes in CDS spreads affect the CBR?; (ii) is there a nexus between CBR and FX rates?; (iii) is there a relationship between CDS spreads and FX rates?; (iv) do the impacts vary over times, frequencies, and quantiles? The empirical outcomes of the study reveal that (i) there is a time-frequency dependency between indicators; (ii) there is a bidirectional link between the CBR and FX rates; between the FX rates and CDS spreads; between the CDS spreads and CBR; (iii) the link exists in most quantiles except for some lower and middle quantiles for some indicators; (iv) the explanatory effects of indicators change according to quantiles; (v) the robustness of the results is validated.

Accordingly, the study contributes in various ways: First of all, the study focuses on Turkey, which has negative outlier conditions compared to its peer emerging countries. Furthermore, it is considered a leading study that examines the dynamic link between three important macroeconomic and financial stability indicators as CBR, CDS spreads, and FX rates simultaneously. It is acknowledged that various studies have examined the link between some of these variables [24,27,32], however, any of these studies have not examined these three variables at the same time. Moreover, Turkey has not been comprehensively explored for these indicators by using high-frequency data. Hence, according to the best knowledge, this is a leading study that examines three indicators simultaneously for the Turkish economy. In addition, the study uses a relatively long period of weekly data from January 2, 2004 to November 12, 2021 as well as applies various time series econometric (WC, QQR, GCQ, TY, and QR) approaches to examine the causality and the impacts between CBR, CDS spreads, and FX rates comprehensively.

Following this section, Section 2 presents the progress of the indicators for Turkey. Section 3 presents conceptual framework and literature review. While section 4 details the methodology, Section 5 presents and discusses the results. Finally, section 6 concludes.

## 2 The progress of the indicators in Turkey

**Fig. 1:**
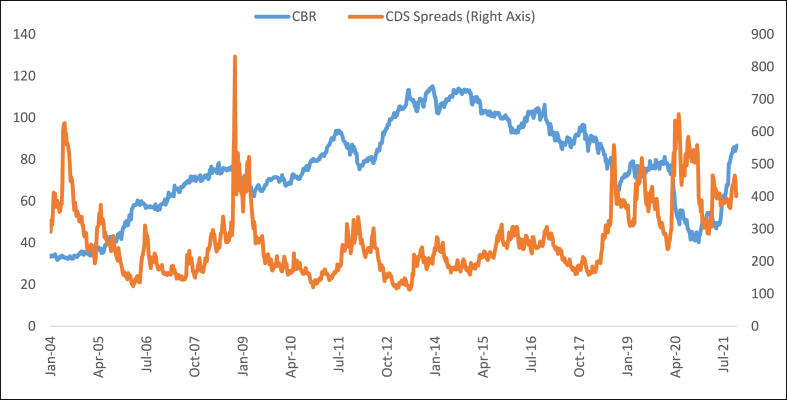
The progress of the CBR and CDS spreads in Turkey.

**Source:** [18,20].

Although CBR, CDS, and FX rates are generally stable in developed country cases, they are much more volatile in emerging countries. Especially, some emerging countries, which take place in BRICST, Emerging-7, MINT, and Fragile Five, have been facing serious volatility. As stated, Turkey is the most negative outlier country in terms of the progress of these indicators. Fig. 1 presents the progress of the CBR and CDS spreads in Turkey.

As Fig. 1 shows, the CBR of Turkey began to decrease at the 2014 year-end. In recent times, the decreasing trend has accelerated and the CBR of Turkey has decreased to the level of the year 2005. With recent measures taken, the CBR of Turkey has been increasing again, but it is still at a low level. However, the CDS spreads of Turkey were quite high in the year 2008 when the global crisis occurred. Except for this, the CDS spreads of Turkey have a relatively short wave until the mid of 2018. After this time, the CDS spreads increase rapidly while the CBR decreases in time. Thus, Fig. 1 implies that a decrease in the CBR may cause an increase in the CDS spreads.

**Fig. 2:**
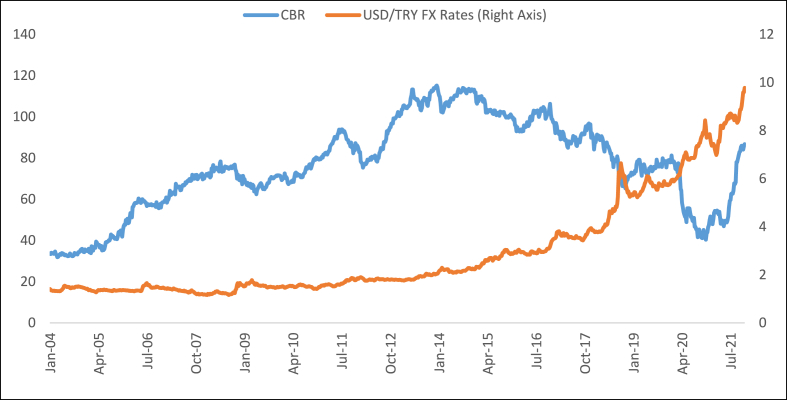
The progress of the CBR and USD/TRY FX rates in Turkey.

**Source:** [20].

Also, Fig. 2 presents the progress of the CBR and USD/TRY FX rates in Turkey.

**Fig. 3:**
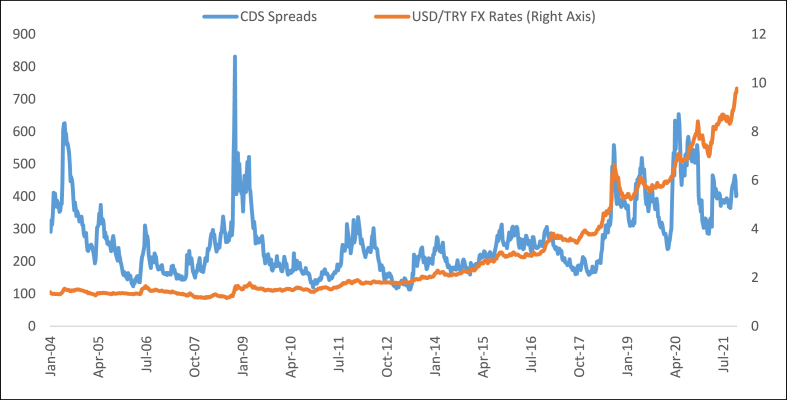
The progress of the CDS spreads and USD/TRY FX rates in Turkey.

**Source:** [18,20].

As Fig. 2 shows, USD/TRY FX rates have been increasing dramatically. Whereas this increase is slow until 2017 end, it has been increasing rapidly after this time. Thus, Fig. 2 implies that a decrease in the CBR may cause an increase in the FX rates. Moreover, Fig. 3 presents the progress of the CDS spreads and FX rates in Turkey.

As Fig. 3 shows, the CDS spreads of Turkey increase while USD/TRY FX rates increase. Hence, Fig. 3 implies that an increase in the CDS spreads may cause an increase in the FX rates. When considering the progress of the CBR, CDS, and FX rates in Turkey altogether, which are presented in detail in Fig. 1, Fig. 2, Fig. 3, either a positive or a negative link can be researched between (i) the CBR and CDS spreads; (ii) the CBR and FX rates (i.e., USD/TRY); (iii) the CDS spreads and FX rates. Hence, causality and impact examinations between these indicators are deserved to be analyzed empirically.

## 3 Conceptual framework and literature review

### 3.1 Conceptual framework

Central banking theory suggests that central banks are among the most important financial institutions and their policies and actions affect economies [33]. According to the central banking theory, the policies of central banks (CB) can be influential on macroeconomic and financial indicators, which makes an effect through monetary policy decisions including the usage of reserves [34]. Thus, CB affects various indicators, such as FX rates [35] and CDS spreads [36].

The CBR has a significant role in international payments as well as in stabilizing FX rates. Countries frequently use CBR in balancing the value of national currencies against FX rates like USD, Euro, and Sterling [13]. Moreover, the CBR can be used to intervene in FX markets when volatility in these markets is too much [15]. However, such interventions can be highly reserve-consuming as can be seen from the experience of Turkey, where billions of USD-based FX reserves are consumed [20]. Especially, the CBR is a significant tool in terms of the aims of monetary policy, which are to provide financial stability as well as price stability [15]. For this reason, the CBR is critical for countries that have a decreasing local currency value, current account deficit, and high FX-denominated debt burden [13,37].

The CDS spreads are also used as insurance for lenders, who are an investor in debt securities of other countries [38]. Besides, the CDS spreads can be used frequently as a country risk indicator because they show how much premium on insurance of default risk of countries is demanded [27]. When the CBR of countries is strong, it can be argued that the default risk of such countries is relatively low. Hence, low-level CDS spreads can be assumed for such countries. Therefore, a negative link is expected between the CBR and CDS spreads in line with the studies of [14,22].

Besides, the FX rates are quite crucial for countries. A big share of international activities is realized with FX payments, especially USD payments. In addition, the FX rates are important in terms of almost all domestic economic activities due to the contingency effect. Furthermore, FX rates can be evaluated as an investment alternative by investors. Hence, a negative link is expected between the CBR and FX rates [17,24]. However, a positive link is expected between the FX rates and CDS spreads as in line with the studies of [16,27,[39], [40], [41]].

### 3.2 Literature review

In the first group [13,24,37], highlights the importance of the CBR for such countries that have a decreasing value of the national currency, current account deficit, and high FX-denominated debt burden. Specifically [13], examine factors that are effective on the CBR [24]. investigate the link between CB gold reserves and sovereign credit risk.

In the second group [14,16,27,38,39], focus on the sovereign risk and CDS spreads. In detail [38], examines the effect of global and local factors on CDS spreads [39]. conclude that reserves are one of the determinants of sovereign risk. Also [14], investigates the role of macroeconomic factors, while [16,27] study the role of global and macroeconomic factors for CDS spreads. Consequently, these studies investigate the link between CDS spreads, and global, and national factors including macroeconomic and financial indicators.

In the third group, various studies handle the FX rates from different perspectives [3,6,12,27,31,42,43]. Current studies in the literature highlight the significance of the FX rates for economies. In this context, some studies focus on the link between FX rates and CBR [17,24], whereas others examine the link between FX rates and country/sovereign risk proxied mainly by CDS spreads [12,26,27,30,31,40,41].

On the other hand, for the case of examination of the three variables altogether, the literature is very weak. There is no study examining the link between these variables altogether according to the best knowledge. On the other hand, it is acknowledged that some studies examine the link between some of these variables, such as CBR and sovereign risk [39]; CDS spreads and FX rates [16]; FX rates and sovereign/country risk [12,30], CBR and sovereign risk [24].

### 3.3 Literature evaluation

The present literature includes various studies examining the link between CBR, CDS, and FX. In these studies, various econometric techniques, such as dynamic conditional correlation, generalized autoregressive conditional heteroskedasticity, generalized method of moments, Granger causality, nonlinear autoregressive distributed lag, panel data analysis, vector autoregression, vector error correction model, are generally performed to examine the link of the indicators with other indicators as well as the link between these indicators. But, based on the best knowledge, no study has examined these three indicators altogether. Also, although there have been adverse developments in these indicators for emerging countries, especially for Turkey, no study has examined Turkey's case comprehensively by including all these three indicators and using high-frequency data. From this perspective, this is considered a gap in the present literature. Considering the gap, the study aims to contribute by inspecting the dynamic link between CBR, CDS spreads, and FX rates simultaneously, using high-frequency data in a relatively long period, and applying various time series econometric approaches (i.e., WC, QQR, GCQ, TY, and QR) to examine the causality and the impact between CBR, CDS spreads, and FX rates comprehensively. Hence, the study provides time, frequency, and quantile varying empirical results for the dynamic link between CBR, CDS spreads, and FX rates.

### 3.4 Hypotheses of the research

By considering the present studies in the literature, the study tests following hypotheses:

⁃ Hypothesis 1: A negative link exists between CBR and CDS.
⁃ Hypothesis 2: A negative link exists between CBR and FX.
⁃ Hypothesis 3: A positive link exists between CDS and FX.

## 4 Methods

### 4.1 Data

Although data for the CBR and USD/TRY FX rates go back until 1988, unfortunately, data for CDS spreads starts from the year 2000. Moreover, Turkey faced two financial crises in 2000 and 2001, and the negative effects of these crises continued during the years 2002 and 2003. By considering these issues, the data started from the year 2004, which is evaluated as a relatively normal year. Hence, all dataset is between January 2, 2004 and November 12, 2021.

Data for the CDS spreads is gathered from Ref. [18]. Also, data for the CBR and USD/TRY FX rates is gathered from Ref. [20]. In this study, a weekly dataset is used because the CBR is announced weekly. Table 1 summarizes the variables.

**Table 1:** Variables.

Variables	Symbols	Descriptions	Units	Data Source
CB Reserves	CBR	CB FX Reserves	Billion USD	[20]
CDS Spreads	CDS	5-Year USD CDS Spreads	Point	[18]
FX Rates	USD/TRY	USD/TRY FX Rates	Point	[20]

### 4.2 Methodological process

**Fig. 4:**
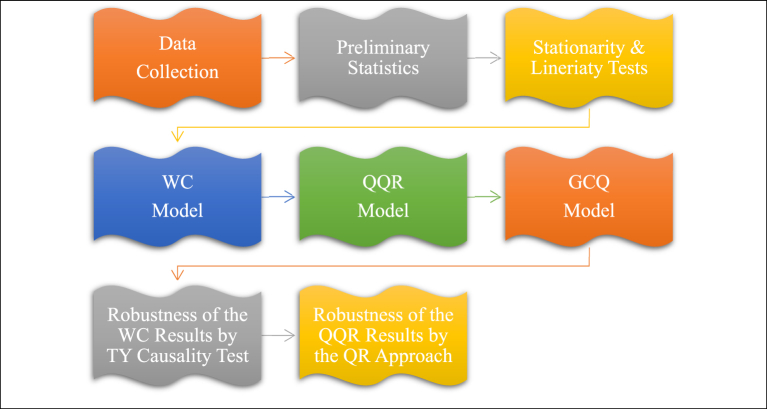
Proposed methodology.

In this study, selected time series econometric models are performed to examine the link between CBR, CDS spreads, and USD/TRY FX rates in Turkey. An eight-step proposed methodology is applied as presented in Fig. 4.

In the empirical analysis;

⁃ The first step of this study is the data-gathering process, in which necessary data is collected from multiple sources.
⁃ The second step is to examine and interpret preliminary statistics of the variables.
⁃ The third step is to examine the stationarity conditions of the variables. So, the Augmented Dickey-Fuller (ADF) test [44] is mainly performed whereas Philip & Perron (PP) test and Zivot & Andrews (ZA) structural break unit root test are included for the robustness of the ADF test results [45,46]. After stationarity, the linearity conditions of the variables are examined, and accordingly, the BDS test [47] is performed. The nonlinear techniques should be used if the results show that the variables are not linear.
⁃ The fourth step is to apply the WC approach by taking into consideration the results of the stationarity and linearity tests. The WC approach captures the link between variables by considering time and frequency [[48], [49], [50], [51]].
⁃ The fifth step is to apply the QQR approach [52]. This approach presents the link between variables at different quantiles by making an impact analysis.
⁃ The sixth step is to apply the GCQ approach [53]. This approach presents the link between variables at different quantiles by presenting a causality analysis.
⁃ The seventh step is to apply the TY causality test to define the causality link between variables as the robustness for the WC approach [54].
⁃ The eighth step is to apply the QR approach as the robustness for the QQR approach [55].

The main advantage of the time series econometric models applied in the study is that they do not require all the variables to be stationary in the same integrated order [49,[56], [57]]. Also, WC, QQR, GCQ, and QR approaches are nonlinear methods, which can present much better results when variables are not linear.

### 4.3 Econometric models

#### 4.3.1 The WC approach

The WC approach captures the dynamic correlation between the variables at different times and frequencies. Hence, it is possible to examine the dynamic link between variable pairs at different frequencies by using WC approach.

A co-movement between two time series (p, q) is examined through applying WC approach that is presented in Eq. (1) [49]:

(1) $$R^{2}\left(a,b\right)=\frac{{\left|N\left(N^{-1}W_{xy}\left(a,b\right)\right)\right|}^{2}}{N\left(N^{-1}{{|W}_{x}\left(a,b\right)|}^{2}\right)N\left(N^{-1}{{|W}_{y}\left(a,b\right)|}^{2}\right)}$$

where $R^{2}$ is WC coefficient ($0\leq R^{2}\;\left(a,.\right)\leq 1$), a denotes location, b denotes frequency, and N denotes time period and the smoothing process. Also, interactions between two time series are determined by WC approach that is specified in Eq. (2) [49]:

(2) $$\pi_{cd}\left(m,n\right)=\tan^{-1}\left(\frac{T\left\{S\left(f^{-1}W_{pj}\left(m,n\right)\right)\right\}}{Q\left\{S\left(f^{-1}W_{pj}\left(m,n\right)\right)\right\}}\right)$$

where Q shows the actual part function, T indicates the imaginary function. In all WC figures, “*the black cone shows the influence area. The warmer colors show a higher degree of dependence between the variables. 0–8 scale shows short-term, 8–64 scale shows medium-term, 64–128 scale shows long-term, and 128–256 scale shows very long-term. 0-0.4 shows low frequency, 0.4-0.6 shows medium frequency, and 0.6-1.0 shows high frequency. The right arrows show a positive correlation while the left arrows show a negative correlation between variables. Right-down and left-up arrows show that the first variable causes the second variable. Also, right-up and left-down arrows show that the second variable causes the first variable*”. Moreover, a footnote is presented to under each WC figures to specify the dependent and independent variables to understand the lead lag relationship.

#### 4.3.2 The QQR approach

The QQR approach examines the impact of the tth quantile coefficient of the independent indicator on the qth quantile of the dependent indicator vice versa.

The QQR approach, which investigates the impact of the independent variable on the dependent variable, can be expressed as Eq. (3) [52]:

(3) $$A_{t}=\beta^{\sigma }\left(C_{t}\right)+\mu_{t}^{\sigma }$$

where B represents the independent variable (i.e., CBR, CDS, FX, in order) and $A_{t}$ denotes dependent variable (i.e., remaining one indicator among CBR, CDS, FX) in the t period, σ denotes $\sigma^{th}$ quantile, and $\mu_{t}^{\sigma }$ is the quantile residual term. Since interactions between the variables are not known a priori, $\beta^{\sigma }$ represents a non-defined function.

#### 4.3.3 The GCQ approach

The GCQ approach examines the causal impact of the tth quantile coefficient of the independent indicator on the qth quantile of the dependent indicator.

The GCQ approach, which searches the impact of the independent variable on the dependent variable, can be expressed as Eq. (4) [53]:

(4) $$Q_{\pi }^{{CO}_{2}}=\left({{CO}_{2}}_{i}|M_{i}^{{CO}_{2}},M_{i}^{GBI}\right)=\lambda_{1}\left(\pi \right)+\lambda_{2}\left(\pi \right){{CO}_{2}}_{i-1}+\eta \left(\pi \right){GBI}_{i-1}+\mu_{t}\psi_{GBI}^{-1}$$

where $\lambda_{1}$, $\lambda_{2}$ and $\mu_{t}$ are re-evaluated based on the highest probability value, and $\psi_{GBI}^{-1}\left(.\right)$ is the inverse of the old conventional conditional distribution function.

## 5 Empirical analysis

### 5.1 Descriptive statistics

Table 2 presents descriptive statistics of the variables that include weekly data from January 2, 2004 to November 12, 2021 and consist of 933 observations.

**Table 2:** Descriptive statistics.

Parameters	CBR	CDS	USD/TRY
Mean	76.11179	265.1458	2.886271
Median	75.68200	239.2810	1.816240
Maximum	115.1440	831.3110	9.774760
Minimum	31.85300	112.8870	1.158520
Standard Deviation	22.08096	110.2764	2.059886
Skewness	−0.233214	1.237267	1.448985
Kurtosis	2.251651	4.384050	3.978951
Jarque-Bera	30.22850	312.5127	363.7370
Probability	0.0000	0.0000	0.0000
Observations	933	933	933

As Table 2 shows, CBR is between USD 31.85 billion and USD 115.14, the CDS spreads are between 112.8 basis points (bps) and 831.31 bps, which imply %1.1 and %8.3 interest rates; and USD/TRY FX rates are between 1.16 and 9.77. Moreover, the standard deviation is 22.08 for the CBR, 110.28 for the CDS spreads, and 2.06 for the USD/TRY FX rates, which reveals that the CDS spreads are quite volatile concerning the CBR and USD/TRY. Moreover, the probability values of the Jarque-Bera test show that the variables are not normally distributed.

### 5.2 Stationarity test

Table 3 presents the results of the ADF, PP, and ZA tests that are used to investigate the stationarity condition of the variables.

**Table 3:** Stationarity test results.

Test	Level/Difference	CBR	CDS	USD/TRY
ADF Test	Level Probability Test Statistic	0.2047 [-2.205121]	0.0054 [-3.629734]	1.000 [-4.476319]
	1st Difference Probability Test Statistic	0.0000 [-6.240190]		0.0000 [-7.362254]
PP Test	Level Probability Test Statistic	0.3149 [-1.937771]	0.0034 [-3.770240]	1.000 [3.479980]
	1st Difference Probability Test Statistics	0.0000 [-32.85473]		0.0000 [-25.51257]
ZA Test	Level Probability Test Statistics Time Breaks	0.003809 [-3.449268] (11/18/2016)	0.000567 [-5.212018] (April 05, 2018)	0.028847 [-1.442383] (03/16/2018)
	**Result**	**I(1)**	**I(0)**	**I(1)**

Notes: ADF Test: Lag length criteria are automatically selected based on the Akaike information criterion (AIC) in the ADF Test; Bartlett kernel in the PP Test; and automatically selected in the ZA Test.

As Table 3 shows, the ADF test results indicate that CBR and USD/TRY are stationary at I(1) and CDS is stationary at I(0). Also, the results of the PP and ZA tests are consistent with the ADF test. Moreover, the results of the ZA test show that the non-stationarity of the CBR and USD/TRY do not result from the structural breaks. Hence, it can be concluded that the CBR is I(1), CDS spreads are I(0), and USD/TRY FX rates are I(1).

### 5.3 Linearity test

After defining the stationarity conditions of the variables, Table 4 presents the results of the linearity conditions that are examined by applying the BDS test.

**Table 4:** Linearity test results.

Dimension	CBR	CDS	USD/TRY
2	0.1966 [0.000]	0.1757 [0.000]	0.2041 [0.000]
3	0.3347 [0.000]	0.2986 [0.000]	0.3469 [0.000]
4	0.4303 [0.000]	0.3809 [0.000]	0.4472 [0.000]
5	0.4959 [0.000]	0.4347 [0.000]	0.5177 [0.000]
6	0.5405 [0.000]	0.4687 [0.000]	0.5675 [0.000]

Notes: The square brackets present the p-values.

The results of the BDS test reveal the nonlinearity structure for the variables. As a result of the examination of the normal distribution, stationarity, and linearity, it can be concluded that linear methods cannot be used for empirical analyses. Therefore, nonlinear methods should be used. Accordingly, this study applies a variety of nonlinear models for empirical examination.

### 5.4 The WC results

**Fig. 5:**
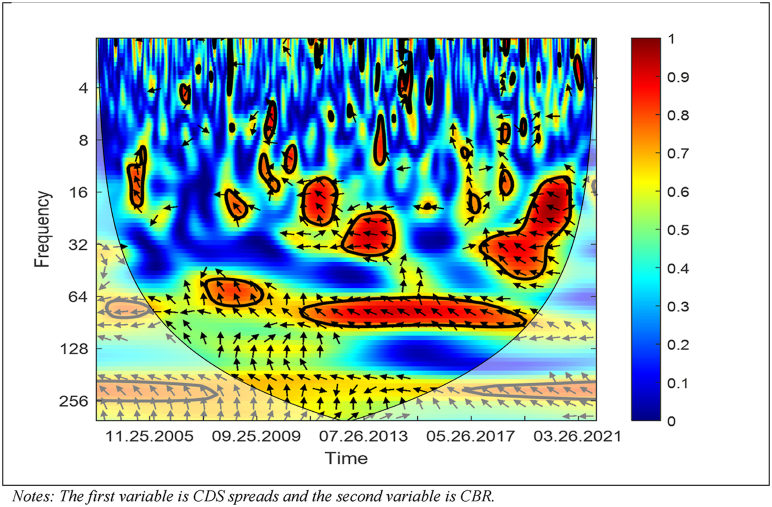
WC between the CDS Spreads and CBR. Notes: The first variable is CDS spreads and the second variable is CBR.

Considering variables’ characteristics, the WC approach is applied first in the context of empirical analysis. Fig. 5 presents the results of the WC approach between the CDS spreads and CBR.

As Fig. 5 shows, in the medium term, the CDS spreads and CBR have a negative association and higher dependence after August 26, 2011 until May 28, 2019. Also, there are some arrows, which are right-up implying that the CBR affects also CDS spreads. Moreover, most of the arrows are left up showing that the CDS spreads cause the CBR. Hence, this figure implies that a decrease in the CDS spreads leads to an increase in the CBR, while a decrease in the CBR leads to an increase in the CDS spreads.

**Fig. 6:**
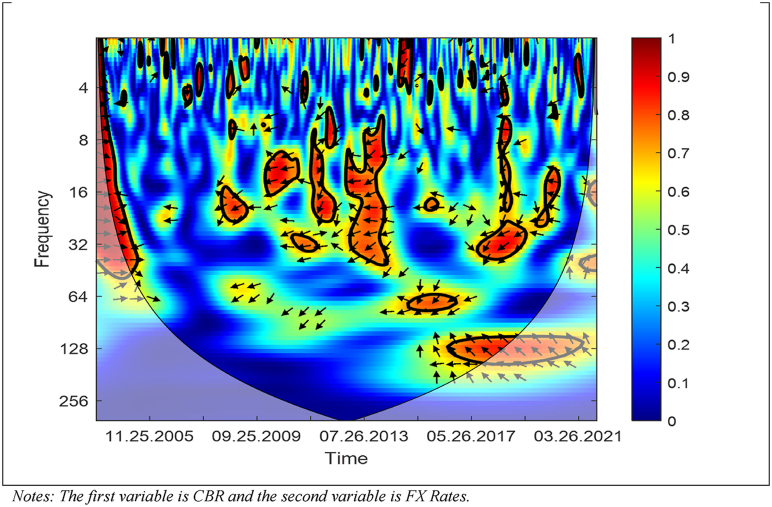
WC between CBR and USD/TRY FX Rates. Notes: The first variable is CBR and the second variable is FX Rates.

Fig. 6 presents the results of the WC approach between the CBR and USD/TRY FX rates.

As Fig. 6 shows, in the medium and long term, the CBR and USD/TRY FX rates have a negative correlation and higher dependence (i) around August 26, 2011, and (ii) after June 26, 2015 until May 28, 2021. Moreover, the majority of arrows are left-up and left-down showing that the CBR cause the USD/TRY FX rates in the middle term while the USD/TRY FX rates cause the CBR in the long term. Hence, the figure shows that a decrease in the CBR leads to an increase in the USD/TRY FX rates, whereas an increase in the USD/TRY FX rates leads to a decrease in the CBR.

**Fig. 7:**
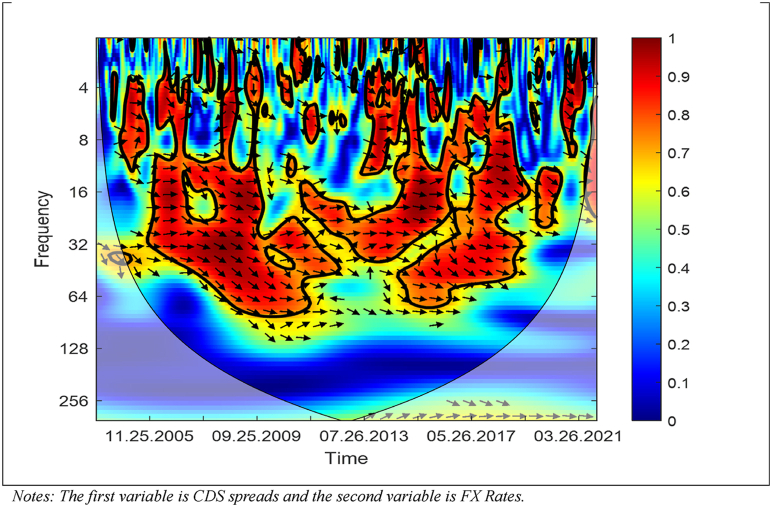
WC between CDS Spreads and USD/TRY FX Rates. Notes: The first variable is CDS spreads and the second variable is FX Rates.

Fig. 7 presents the results of the WC approach between the CDS Spreads and USD/TRY FX rates.

As Fig. 7 shows, in the short and medium term, the CDS spreads and USD/TRY FX rates have a positive link and higher dependence (i) around August 26, 2011, and (ii) after June 26, 2015 until May 28, 2021. Moreover, the majority of arrows are right-down while a few of them are right-up representing that the CDS spreads cause the USD/TRY FX rates while the USD/TRY FX rates cause the CDS spreads. Hence, the figure shows that an increase in the CDS spreads leads to an increase in the USD/TRY FX rates, while an increase in the USD/TRY FX rates also leads to an increase in the CDS spreads.

The outcomes, which are obtained from the WC approach, show that there is time-frequency dependency between the CBR, CDS spreads, and USD/TRY FX rates in mainly medium-term and high frequencies. Moreover, a bidirectional link between the variables exists. Furthermore, the outcomes reveal that the CDS spreads drive the CBR after August 26, 2011 until May 28, 2021; the CBR and USD/TRY FX rates drive each other (i) around August 26, 2011, and (ii) after June 26, 2015 until May 28, 2021; the CDS spreads and USD/TRY FX rates drive each other (i) around of August 26, 2011, and (ii) after June 26, 2015 until May 28, 2021.

### 5.5 The QQR results

**Fig. 8:**
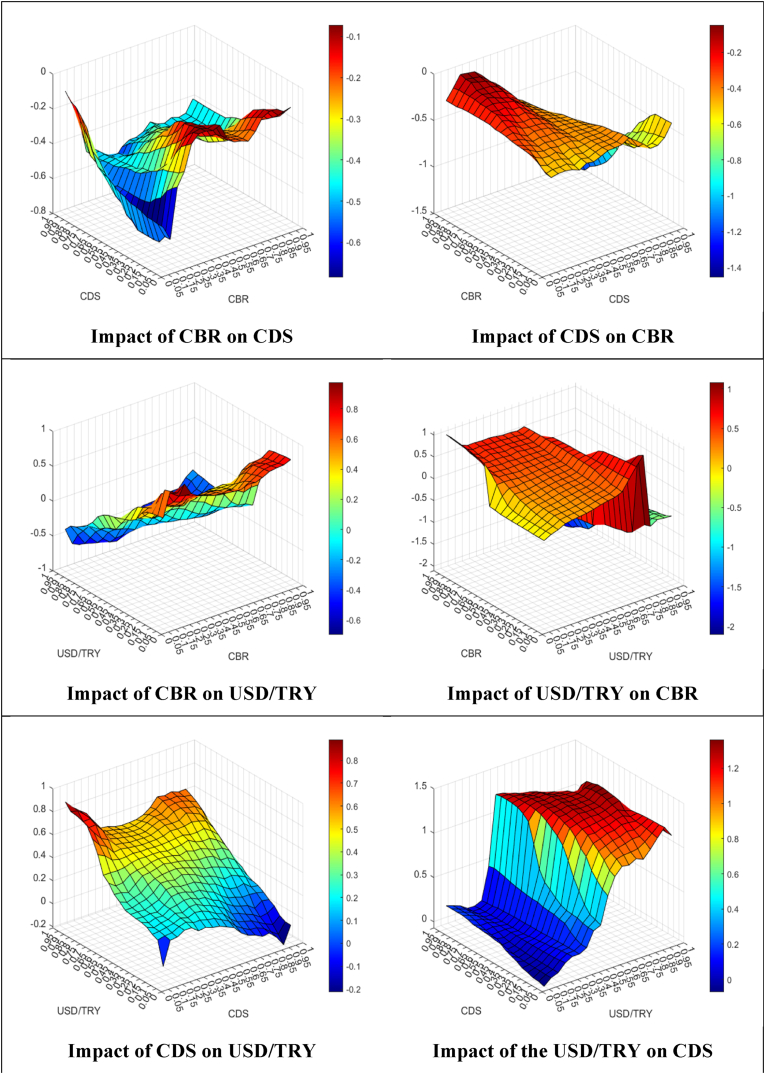
The QQR results.

After the GCQ approach, the QQR approach is applied to examine the power (i.e., impact) of the link between the indicators at the different levels (i.e., quantiles). Fig. 8 shows the results of the QQR approach by using the impact of the tth quantile coefficient of the independent indicator on the qth quantile of the dependent indicator and vice versa.

The effects of the CBR on the CDS spreads are negative in all quantiles. However, the effects are much stronger in the high quantiles of the CBR and the low quantiles of the CDS spreads. Besides, the effects are high in the low quantiles of the CBR and high quantiles of the CDS spreads. Similarly, the effects of the CDS spreads on the CBR are negative and quite stronger in all quantiles. The effects are stronger in the low quantiles of the CDS spreads and the high quantiles of the CBR.

The influences of the CBR on the USD/TRY FX rates are negative in almost all quantiles of the CBR and middle and high quantiles of the USD/TRY. On the other hand, the influences of the CBR on the USD/TRY FX rates are positive in high quantiles of the CBR and low quantiles of the USD/TRY. On the opposite side, the effects of the USD/TRY FX rates on the CBR are generally positive and pretty stronger at the middle quantiles of the USD/TRY FX rates and all quantiles of the CBR. On the other hand, the effects are negative in high quantiles of the USD/TRY FX rates and high quantiles of the CBR.

The impacts of the CDS spreads on the USD/TRY FX rates are negative at higher quantiles of the CDS spreads and low quantiles of the USD/TRY. On the other hand, the impacts are positive at the middle quantiles of the CDS spreads and the middle quantiles of the USD/TRY. Also, the impacts are much stronger at lower quantiles of the CDS spreads and high quantiles of the USD/TRY. On the opposite side, the impacts of the USD/TRY FX rates on the CDS spreads are generally positive and rather stronger at the middle and high quantiles of the USD/TRY FX rates and all quantiles of the CDS spreads. On the other hand, the effects are still positive but weak at low quantiles of the USD/TRY FX rates and all quantiles of the CDS spreads.

### 5.6 The GCQ results

After examining time and frequency dependency between the variables via the WC approach and investigating the causality between variables via the TY causality test, causality in quantiles is also investigated by applying the CCQ approach. Table 5 shows the results of the GCQ approach.

**Table 5:** The GCQ results.

Causality Path	Quantiles																		
	0.05	0.10	0.15	0.20	0.25	0.30	0.35	0.40	0.45	0.50	0.55	0.60	0.65	0.70	0.75	0.80	0.85	0.90	0.95
CBR > CDS	0.00	0.00	0.00	0.00	0.00	0.00	0.00	0.79	0.30	0.00	0.00	0.00	0.00	0.00	0.00	0.00	0.00	0.00	0.01
CDS > CBR	0.00	0.74	0.00	0.00	0.00	0.00	0.00	0.00	0.00	0.22	0.54	0.02	0.00	0.00	0.00	0.00	0.00	0.00	0.00
CBR > USD/TRY	0.00	0.00	0.00	0.00	0.00	0.00	0.00	0.09	0.56	0.33	0.00	0.00	0.00	0.00	0.00	0.00	0.00	0.00	0.00
USD/TRY > CBR	0.00	0.75	0.00	0.00	0.00	0.00	0.00	0.00	0.00	0.24	0.61	0.02	0.00	0.00	0.00	0.00	0.00	0.00	0.00
CDS > USD/TRY	0.00	0.00	0.00	0.00	0.00	0.00	0.00	0.06	0.29	0.11	0.00	0.00	0.00	0.00	0.00	0.00	0.00	0.00	0.00
USD/TRY > CDS	0.00	0.00	0.00	0.00	0.00	0.00	0.00	0.82	0.30	0.00	0.00	0.00	0.00	0.00	0.00	0.00	0.00	0.00	0.01

Note: Numbers represent p-values.

According to Table 5, almost at all quantiles except some, there is a Granger causality between the CBR and CDS spreads; between the CBR and USD/TR FX rates; and between the CDS spreads and USD/TRY FX rates at a 5% level of statistical significance. There are bidirectional causalities between variables at quantiles that are higher than 0.55. On the other hand, the causality does not exist between variables at lower quantiles (i.e., 0.10), and some middle quantiles (i.e., 0.40, 0.45, 0.50, 0.55) for some variable pairs. As a whole, these results reveal that there is bidirectional Granger causality between the variables.

### 5.7 Robustness

#### 5.7.1 The TY causality results

As the robustness of the WC approach, the TY causality test is applied. By considering that the variables have different integrated orders, the TY causality test is performed to examine the dynamic link between the variables. In the TY causality test, lag length (k) and maximum cointegration degree (d_max_) should be stated in the first step. According to Annex 3, the maximum cointegration degree (d_max_) is determined as one. Also, lag length (k) is defined as ten because the AIC and Final prediction error have lower values than the Schwarz information criterion and Hannan-Quinn information criterion, implying that this lag length (i.e., ten lengths) is much more appropriate than the other (i.e., two lengths) for financial time series [58]. Hence, the estimation degree (k + d_max_) is defined as eleven.

In the second step, the TY causality test is performed under the vector autoregressive approach to examine the link between the variables by using the estimation degree defined in the first step. Table 6 shows the results of the TY causality test.

**Table 6:** TY causality test.

Causality Paths	(k + d_max_)	X^2^ Test Statistics for k [p-values]	Result
CBR => CDS	11	13.75296 [0.0578]**	Causality
CDS => CBR	11	27.12897 [0.0009]*	Causality
CBR => USD/TRY	11	14.84842 [0.0378]*	Causality
USD/TRY => CBR	11	24.93690 [0.0019]*	Causality
USD/TRY => CDS	11	24.26493 [0.0024]*	Causality
CDS => USD/TRY	11	155.1302 [0.0000]*	Causality

Notes: * and ** show 5% and 10% significance levels, respectively.

As Table 6 presents, there are bidirectional causalities between the CBR and CDS spreads; between the CBR and USD/TRY FX rates; between CDS spreads and USD/TRY FX rates. The results are rather important because they provide details regarding the causality link between the CBR, CDS spreads, and USD/TRY FX rates for Turkey.

#### 5.7.2 The QR approach

**Fig. 9:**
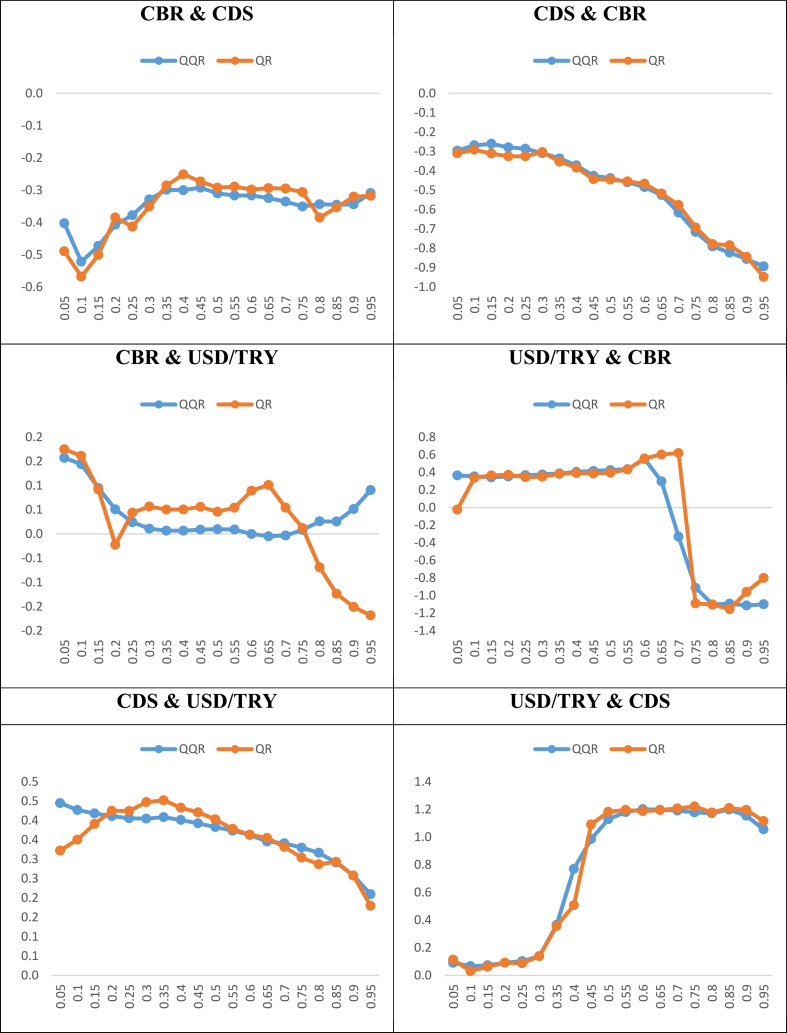
Robustness results between the QQR and QR approaches.

Lastly, as the robustness of the QQR approach, the QR approach is applied. Fig. 9 shows the comparison of the coefficients for different quantiles that are obtained from the QQR and QR approaches.

As Fig. 9 presents, it can be seen that the correlation between the QQR and QR approaches is quite similar and statistically significant. Hence, the results show that the QQR results are robust according to the QR approach results.

### 5.8 Discussion

The WC results reveal that there is a bidirectional link between variables. Also, the QQR and GCQ approaches show that the links between the variables change according to the quantiles. To sum up, a negative link is largely found between the CBR and CDS spreads; and between the CBR and USD/TRY FX rates, whereas a positive link is mostly found between the CDS spreads and FX rates. Also, by considering the results of the multi tests applied (i.e., WC, QQR, GCQ, TY, and QR approaches), the alternative models (i.e., TY and QR approaches) validate the robustness of the main models’ results (WC, QQR, and QR approaches).

Based on the empirical findings, the results confirm the hypotheses, which are examined in the study. The outcomes of the study are generally consistent with the studies [10,11,27] for CDS [12,31], for FX, and [[13], [14], [15], [16],^19^,^24^,^26^,^27^] for CBR. Hence, the results are important because they provide details regarding the dynamic link between CBR, CDS spreads, and USD/TRY in Turkey. However, different from the current studies, this study reveals a link between CBR, CDS spreads, and USD/TRY and it varies according to times, frequencies, and levels (i.e., quantiles) of the variables.

Based on the results, it can be expressed that these three indicators are significant predictors for each other in Turkey. Therefore, Turkey should focus on these indicators altogether to keep them at minimum levels and prevent negative effects from one to others. Thus, by considering the analysis results, various policy proposals can be recommended below.

The results of the analysis can be read that the priority for Turkey should be dealing with the CBR, CDS spreads, and FX rates in a harmonious way. Accordingly, Turkey should focus firstly on decreasing country risk (e.g., CDS spreads in the study) so that the CBR can be increased by considering the bidirectional link between these variables. In this context, structural reforms to manage political, economic, and financial risks are crucial. By taking necessary precautions, Turkey can decrease CDS spreads. Besides, by putting some regulatory legislation into effect, the CBR can be increased rapidly in Turkey. In this context, the results support the proposals of applying a reserve tax to financial institutions rather than required reserves and transferring the Treasury's share in the profit of CB to reserves rather than the Treasury as in line with the studies of [15,33].

By achieving a decrease in the CDS spreads, Turkey can also achieve success in making the FX rates (i.e., USD/TRY FX rates) stable by benefiting from the bidirectional link between the CBR and FX rates. Likewise, Turkey can have some positive progress in the CDS spreads in this way because there is also a bidirectional link between the FX rates and CDS spreads.

Turkey can also take other global, macroeconomic, and financial indicators as well as political issues into consideration so that more comprehensive and successful policies can be developed and implemented on the CBR, CDS spreads, and FX rates. In this context, it must be stated that countries including Turkey should consider their national conditions and economic structures in developing and implementing policies. Moreover, necessary precautions should be timely taken without any delay. Otherwise, precautions may cause adverse effects rather than making a positive contribution.

Lastly, decision-makers should consider using much higher-frequency data (i.e., daily or hourly data) and can develop much more comprehensive policies by using all macroeconomic and financial information, which is not publicly available. Even, it can be helpful for emerging countries, which are similar to Turkey, to position the CBR, CDS spreads, and FX rates as macro-prudential issues and put them at a very high level in the management of economies in a harmonious way.

## 6 Conclusion

The study investigates the dynamic link between CBR, CDS spreads, and FX rates in Turkey. Turkey is selected because of its negatively outlier condition in terms of these indicators. To the best knowledge, this link has not been comprehensively examined for Turkey although it is one of the most significant emerging economies. Considering the aim of the study, by implementing a series of time series econometric models, and covering a relatively high frequency (i.e., weekly) data from January 2, 2004 to November 12, 2021, a comprehensive examination is done to investigate the aforementioned link between the variables by benefiting from the Turkey case.

The results of the empirical examination reveal that there is a time-frequency dependency between the indicators; a bidirectional link exists between the CBR and FX rates, between the FX rates and CDS spreads, and between the CDS spreads and CBR; the link exists in most quantiles except for some lower and middle quantiles for some indicators; explanatory effects of the indicators on each other change according to the quantiles; and the results are validated. The empirical outcomes are robust and generally consistent with the pre-expectations, hypotheses, and current literature. Hence, the study sheds light on the decision-makers in emerging countries to develop policies to achieve macroeconomic and financial stability through CBR, CDS, and FX.

Furthermore, the results signify the importance of the CBR for the FX rates; the FX rates for the CDS spreads; the CDS spreads for the CBR. Based on the outcomes of the study, various policy proposals, such as positioning the CBR, CDS spreads, and FX rates as a macro-prudential concern, dealing with these indicators in a harmonious way, implying reserve tax, transferring the share of the Treasury in the profit of central bank to reserves, and focusing on decreasing country risk, are recommended. Likewise, country-specific policy customization is highly recommended due to the fact that economic realities and structures of countries can vary at the country-specific level although they have been in the same country groups or peer groups. Hence, it can be stated that other emerging countries, which have volatile progress in these indicators, can benefit from Turkey by taking necessary measures.

This study contributes a various ways. Firstly, the study focuses on Turkey, which has negative outlier conditions compared to the peer emerging countries. Secondly, it is a leading study, which scrutinizes the dynamic link between CBR, CDS spreads, and FX rates simultaneously. Thirdly, Turkey has not been comprehensively explored for these indicators by using high-frequency data. Hence, according to the best knowledge, this is a pioneering study, which examines three indicators simultaneously for the Turkish economy. In addition, the study uses relatively long weekly data from January 2, 2004 to November 12, 2021 as well as applies various time series econometric (WC, QQR, GCQ, TY, and QR) approaches to examine the causality and the impacts between CBR, CDS spreads, and FX rates comprehensively. On the other hand, the two constraints for the study can be spelled out; (i) the scope of the study consists of solely Turkey. Therefore, more emerging countries that have adverse progress in terms of the indicators can be examined in future studies so that the literature can benefit from a comparative examination; (ii) this study performs only time series econometric analysis. However, utilizing other econometric techniques, such as partial wavelet, multivariate wavelet, machine learning algorithms, and rolling window causality, new studies can contribute to the existing literature as well.

## Funding statement

We acknowledge the funding from Gulf University for Science and Technology.

## Declaration of competing interest

The authors declare that they have no known competing financial interests or personal relationships that could have appeared to influence the work reported in this paper.

## Figures and Tables

**Annex 1:**
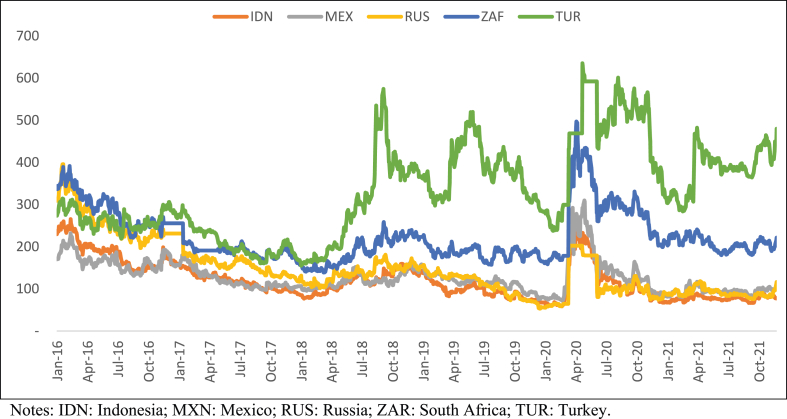
Progress of the CDS Spreads in Emerging Countries Notes: IDN: Indonesia; MXN: Mexico; RUS: Russia; ZAR: South Africa; TUR: Turkey. **Source:** Bloomberg (2021).

**Annex 2:**
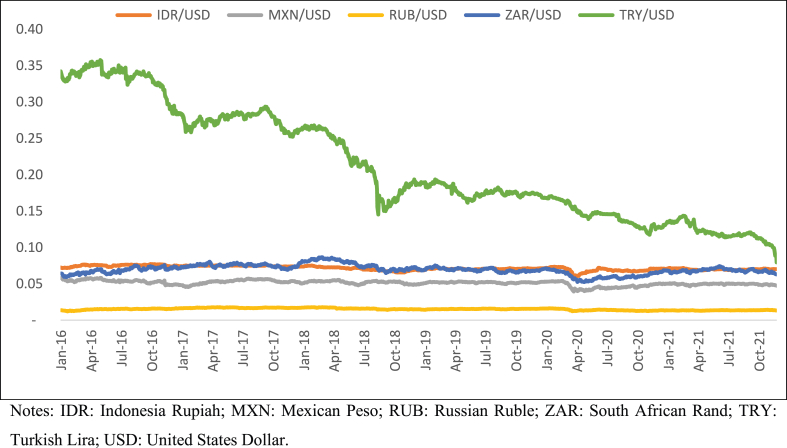
Progress of the National Currencies against USD in Emerging Countries Notes: IDR: Indonesia Rupiah; MXN: Mexican Peso; RUB: Russian Ruble; ZAR: South African Rand; TRY: Turkish Lira; USD: United States Dollar. **Source:** Bloomberg (2021).

**Annex 3:** Lag Length Selection

Lag	LogL	LR	FPE	AIC	SC	HQ
0	−11446.78	NA	12612240	24.86381	24.87953	24.86981
1	−4893.668	13049.31	8.493363	10.65292	10.71579	10.67691
2	−4782.121	221.3988	6.797801	10.43023	10.54026*	10.47221*
3	−4777.191	9.751732	6.858168	10.43907	10.59625	10.49905
4	−4760.898	32.12752	6.750420	10.42323	10.62757	10.50120
5	−4747.413	26.49957	6.685040	10.41349	10.66498	10.50946
6	−4740.335	13.86495	6.713040	10.41767	10.71631	10.53162
7	−4731.876	16.51478	6.720990	10.41884	10.76464	10.55079
8	−4727.877	7.780533	6.794466	10.42970	10.82265	10.57964
9	−4721.415	12.52970	6.832138	10.43521	10.87532	10.60315
**10**	**−4693.137**	54.65309*	6.552154*	10.39335*	**10.88061**	**10.57928**
11	−4689.572	6.866232	6.630109	10.40515	10.93957	10.60908
12	−4681.740	15.03460	6.647141	10.40769	10.98926	10.62961

Notes: LR: Sequential modified LR test statistic; FPE: Final prediction error; AIC: Akaike information criterion; SC: Schwarz information criterion; HQ: Hannan-Quinn information criterion.
